# Utilization of Walnut Male Flowers (*Juglans regia* L.) as a Functional Ingredient in Biscuits: Impacts on Dough Rheology, Nutritional Composition, Glycemic Response, and Sensory Attributes

**DOI:** 10.1002/fsn3.71510

**Published:** 2026-01-30

**Authors:** Guoshuang Qin, Yajing Li, Jian Sun, Zhenxing Wang, Jinggui Nie, Kin Weng Kong, Amin Ismail, Yongyu Si, Xuechun Zhang

**Affiliations:** ^1^ Key Laboratory for Conservation and Utilization of In‐Forest Resource, College of Biological Science and Food Engineering Southwest Forestry University Kunming China; ^2^ Guangxi Key Laboratory of Fruits and Vegetables Storage‐Processing Technology, Guangxi Academy of Agricultural Sciences Nanning China; ^3^ Department of Molecular Medicine, Faculty of Medicine Universiti Malaya Kuala Lumpur Malaysia; ^4^ Department of Nutrition, Faculty of Medicine and Health Sciences Universiti Putra Malaysia (UPM) Serdang Malaysia; ^5^ Department of Anesthesiology Second Affiliated Hospital of Kunming Medical University Kunming China

**Keywords:** biscuits, functional ingredient, male walnut flowers, predicted glycemic index, sensory evaluation, texture profile

## Abstract

Walnut male flowers (WFs), an underutilized by‐product of walnut ( *Juglans regia* L.) processing, are rich in nutrients and contain bioactive compounds with potential health benefits. This study examined the effects of partially substituting wheat flour with WFs (0%, 1%, 3%, 5%, 7%, and 10% w/w) in biscuit formulations on dough rheology, nutritional profile, texture, microstructure, glycemic response, and sensory characteristics. The addition of WFs significantly increased both the storage modulus (G′) and the loss modulus (G″) of the dough, indicating an improvement in its viscoelastic properties. Nutritional composition analysis revealed elevated protein, ash, and dietary fiber contents, accompanied by reduced total sugar and soluble carbohydrate levels. Texture profile analysis revealed decreased hardness and increased crispness, supported by scanning electron microscopy (SEM) observations of a more porous microstructure. The predicted glycemic index (*p*GI) decreased by up to 16% with 10% WFs substitution, which may be due to dietary fiber–mediated starch encapsulation and phenolic inhibition of α‐glucosidase activity. Sensory evaluation indicated that biscuits containing 5% WFs received the highest overall acceptability, achieving a balance of desirable color, texture, and flavor. The results indicate the potential of WFs as a functional ingredient for developing low–glycemic index baked goods and support the sustainable use of agricultural by‐products.

## Graphical abstract

This study investigated walnut male flowers (WFs) as a functional ingredient in biscuits. Replacing wheat flour with 0%–10% WFs enhanced dough viscoelasticity, increased protein and fiber content, and reduced the predicted glycemic index by up to 16%. Sensory evaluation identified 5% WFs as optimal, balancing improved nutritional profile, desirable texture, and high acceptability. The research supports the valorization of this agricultural by‐product for developing healthier, low‐glycemic baked goods.

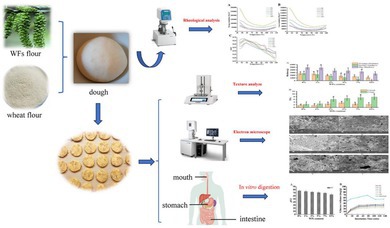

**Abbreviations**
DNS: 3.5‐dinitro salicylic acid
G′: storage modulus
G″: loss modulus
*p*GI: predicted glycemic index
TDF: total dietary fiber
TPA: textural properties analysis
WFs: walnut flowers
WHC: water‐holding capacity

## 1 Introduction

Walnut ( *Juglans regia* L.), a member of the Juglandaceae family, is an economically significant tree nut with a global annual production exceeding 4 million metric tons, making China the leading producer (IndexBox 2025). Recognized for its high nutritional value, walnut is among the four most widely consumed nuts worldwide. Beyond the kernels, walnut processing by‐products, such as the pellicle and male flowers (WFs), have also been reported to possess considerable nutritional and health‐promoting properties (Cao et al. 2024; Zhao et al. 2024). Although the yield of WFs is comparable to that of the nuts, a lack of effective processing technologies results in less than 10% utilization, leading to significant resource wastage (Zhang et al. 2018).

WFs have been traditionally used as a culinary ingredient in various dishes, including salads, stir‐fries, and stews. Nutritional analyses indicate that WFs are rich in protein (23%) and dietary fiber (46%), and contain notable levels of minerals (Ni et al. 2022). Research shows that WFs contain abundant natural bioactive compounds, particularly polyphenols and flavonoids, which are associated with various biological activities, including antioxidant, antihyperglycemic (e.g., via inhibition of α‐glucosidase and α‐amylase), antimicrobial, and anti‐inflammatory effects (Alongi et al. 2019; Muzaffer and Paul 2018; Ru et al. 2022; Zhang et al. 2017). Our preliminary findings suggest that WFs maintain significant inhibitory activity against α‐glucosidase and α‐amylase following In vitro simulated gastrointestinal digestion and fecal fermentation, underscoring their potential hypoglycemic properties (Jin et al. 2022). These phytochemicals underscore the potential of WFs as a functional food ingredient that offers multiple health benefits. However, despite their promising properties, the application of WFs within the food industry remains limited, with few examples of their use as a food ingredient. This underutilization contrasts sharply with their considerable development potential.

Traditional biscuits, characterized by high sugar content, high caloric density, and a high GI, are unsuitable for individuals with diabetes or obesity. Although low‐sugar and low‐calorie biscuit variants are commercially available, they possess significant limitations. While representing an improvement over traditional options, these alternatives often introduce new challenges related to sensory quality and nutritional balance (Ashwath Kumar et al. 2019; Giarnetti et al. 2015; Yong et al. 2023). For example, to reduce sugar and calorie content, formulations frequently incorporate ingredients like whole wheat flour and dietary fiber, which can result in a coarse texture and diminished flavor. To compensate for these sensory drawbacks, fat content may be increased to improve palatability. Furthermore, such products often remain inadequately fortified with protein, vitamins, and minerals, and may rely on artificial flavors or sweeteners, thereby compromising their overall nutritional quality. These shortcomings have driven research into reformulating biscuits by incorporating natural, plant‐based ingredients, particularly low‐cost, nutrient‐rich agricultural by‐products, to enhance sensory properties, nutritional profiles, and potential health benefits. For instance, incorporating coffee extract residue has been shown to enhance total phenolic and flavonoid content as well as antioxidant capacity (Han and Lee 2021). Similarly, the use of goji berry ( *Lycium barbarum* ) processing by‐products (pulp, skin, and seeds) has been reported to improve both the nutritional quality and textural properties of biscuits (Bora et al. 2019). Grape pomace incorporation has been reported to increase protein and fiber content while enhancing flavor and antioxidant activity (Theagarajan et al. 2019). Furthermore, sugarcane bagasse has been found to improve biscuit texture and crispness (Oladunjoye et al. 2021). Given the favorable nutritional and bioactive profile of WFs, these precedents support its promising potential as a functional ingredient in baked goods, particularly biscuits.

Based on this background, this study aimed to incorporate WFs into biscuit formulations and evaluate the impact of different substitution levels on the dough properties, texture, nutritional composition, and glycemic response of the final product. This work responds to increasing consumer interest in health‐promoting, convenient, and palatable food options. To our knowledge, this is the first report on the utilization of WFs in biscuit production. The findings aim to promote the use of WFs in the food industry and provide insights for developing baked goods with improved nutritional value and enhanced environmental sustainability.

## 2 Materials and Methods

### 2.1 Materials and Reagents

Walnut male flowers (WFs) were collected from the Santai‐variety in Da Yao County, Yunnan Province, China. Upon arrival, samples were immediately transported to the laboratory at 4°C and stored at −80°C to preserve heat‐labile phenolic compounds. After removal of stamens, the flowers were oven‐dried at 40°C to minimize flavonoid degradation. The dried material was then pulverized and sieved through a100‐mesh sieve (≤ 150 μm) for subsequent use. Low‐gluten wheat flour, butter, fresh whole eggs, and powdered sugar were purchased from a local supermarket in Kunming, China. α‐Amylase (≥ 5000 U/mg), pepsin (≥ 2500 U/mg), and trypsin (≥ 10,000 U/mg) were obtained from Shanghai Yuan Ye Biotechnology Co. Ltd. (China). All other chemicals and reagents used were of analytical grade.

### 2.2 Biscuit Preparation

Biscuit formulations were adapted from the method described by Jia et al. (2020) methods. The control dough formulation consisted of 180.0 g low‐gluten wheat flour, 50.0 g butter, 36.0 g liquid whole egg, and 40.0 g powdered sugar. Wheat flour was substituted with WFs powder at levels of 0%, 3%, 6%, and 10% (w/w, flour basis). The mixed dough was rested at −20°C for 40 min. A portion of the dough was reserved for analysis, while the remainder was sheeted to a uniform thickness of 5 mm and cut using a circular mold. The shaped dough pieces were placed on a perforated baking tray and baked in a conventional oven at 170°C for 17 min. After baking, the biscuits were cooled at room temperature for 10 min and subsequently stored in sealed polyethylene bags until analysis.

### 2.3 Rheological Analysis

The rheological properties of the dough were evaluated using an MCR302 rheometer (Anton Paar, Germany) according to the method of Wang et al. (2019). Measurements were performed using parallel plates (40 mm diameter, 1 mm gap). A solvent trap cover was used to minimize water evaporation during testing. After loading, the dough sample was allowed to equilibrate for 5 min prior to measurement. The storage modulus (G′), loss modulus (G″), and loss tangent (tan δ = G″/G′) were determined at a fixed frequency of 1 Hz and a strain of 0.5% while heating from 45°C to 120°C at a rate of 5°C/min.

### 2.4 Water‐Holding Capacity (WHC)

WHC was determined according to the method described by Jia et al. (2020). Briefly, 0.5 g of ground biscuit sample was dispersed in 10.0 mL of distilled water. The mixture was equilibrated in a water bath at 37°C for 1 h, followed by centrifugation at 4800 × *g* for 10 min. The resulting precipitate was collected and weighed to obtain the wet weight (WW). It was then dried in a forced‐air oven at 100°C until a constant weight was achieved (dry weight, WD). The WHC was calculated using Equation (1):

(1) $$\mathrm{WHC}\;\left(g/g\right)=\frac{\left(\mathrm{WW}-\mathrm{WD}\right)}{\mathrm{WD}}$$

### 2.5 Texture Profile Analysis

Texture parameters (hardness and crispness) were evaluated using an XT Plus texture analyzer (Stable Micro Systems, Surrey, UK) following the method of Dong et al. (2022). A 5 mm cylindrical probe was used under the following conditions: pre‐test speed of 1.0 mm/s, test speed of 1.0 mm/s, post‐test speed of 10.0 mm/s, and a trigger force of 20 g. Each biscuit sample was measured in triplicate, and average values were calculated.

### 2.6 Scanning Electron Microscopy (SEM)

The microstructure of the biscuits was examined using a scanning electron microscope (MIRA LMS, TESCAN Ltd., Czech Republic). Prior to imaging, samples were sputter‐coated with gold for 60 s at 10 mA using an SC7620 coater (Quorum Technologies Ltd., UK) to ensure sufficient electrical conductivity. Images were acquired at an acceleration voltage of 15 kV.

### 2.7 Nutritional Composition Analysis

The nutritional composition was analyzed using standard AOAC methods with minor modifications (Han and Lee 2021). Ash content was determined according to AOAC method 923.03, and total lipid content was assessed using AOAC method 920.39. Protein content was quantified by the Kjeldahl method (AOAC 920.87), with total protein calculated as total nitrogen × 6.25. Total dietary fiber was analyzed by the enzymatic‐gravimetric method (AOAC 985.29) and calculated as the residue weight minus ash and protein contents. Total carbohydrate content was determined by difference, calculated as 100% minus the sum of moisture, ash, protein, and lipid contents.

### 2.8 Glycemic Index Assay

The glycemic index (GI) was determined according to the method of Xie et al. (2020) with slight modifications. Briefly, 1.0 g of ground biscuit sample was hydrated with 10 mL of distilled water. Then, 10 mL of simulated saliva containing α‐amylase was added, and the pH was adjusted to 7.0. The mixture was incubated at 37°C for 15 min, followed by centrifugation at 8000 × *g* for 10 min. The supernatant was collected as the oral digestion fraction.

The residue was reconstituted in 10 mL of distilled water and mixed with an equal volume of simulated gastric juice. After adjusting the pH to 2.0, gastric digestion was simulated by incubation at 37°C for 1 h. The mixture was then centrifuged at 8000 × *g* for 10 min, and the supernatant was collected as the gastric digestion fraction.

The remaining residue was resuspended in 10 mL of distilled water and combined with an equal volume of simulated intestinal fluid (containing trypsin, 30 mg bile salts, 54 mg NaCl, and 6.5 mg KCl). The pH was adjusted to 7.0, and the mixture was incubated at 37°C for 2 h to simulate the intestinal phase. After centrifugation at 8000 × *g* for 10 min, the supernatant was filtered through a 0.22 μm membrane to obtain the intestinal digestion fraction.

Glucose concentration in the digestion supernatants was quantified using the 3,5‐dinitrosalicylic acid (DNS) method, with reference to a glucose standard curve (Jiang et al. 2022). Glucose release kinetics were plotted as a release‐time curve, and the area under the curve (AUC) was calculated for each sample. The hydrolysis index (HI) was determined by normalizing the sample AUC against that of white bread. The predicted glycemic index (pGI) was calculated using the equation: pGI = 39.71 + 0.549 × HI (Klunklin and Savage 2018).

### 2.9 Sensory Evaluation

Sensory evaluation was conducted using a 9‐point hedonic scale (1 = dislike extremely, 5 = neither like nor dislike, 9 = like extremely) in accordance with ISO 8589:2007. The study protocol was approved by the Ethics Committee of Southwest Forestry University (Approval No. SWFU‐2023026) and was conducted in accordance with the Declaration of Helsinki. Written informed consent was obtained from all participants prior to the study.

A panel of 20 volunteers (10 male and 10 female, aged 20–30 years) was recruited based on the following criteria: non‐smokers, regular consumers of baked goods, and no history of food allergies or intolerances. Prior to evaluation, all panelists received 2 h of training in sensory attribute recognition according to ISO 8589:2007. Evaluations were conducted in a standardized sensory analysis room under controlled environmental conditions. The assessed attributes included color, texture, flavor, aroma, and overall acceptability. Biscuit samples were presented to panelists in a randomized order one day after baking.

### 2.10 Color Analysis

Surface color was measured using a colorimeter (Model NH310, Shenzhen 3NH Technology Co. Ltd., China). The instrument was calibrated with a standard white tile prior to each measurement session. Color parameters, including L* (lightness), a* (redness/greenness), b* (yellowness/blueness), and C* (chroma), were recorded. Measurements were performed in triplicate, and the mean values were calculated for each sample.

### 2.11 Statistical Analysis

All measurements were conducted in triplicate, and data are presented as mean ± standard deviation (SD). Statistical analyses were performed using SPSS software (version 26.0; IBM Corp., Armonk, NY, USA) and Origin 2018 (OriginLab Corporation, Northampton, MA, USA). The significance of differences among group means was assessed by one‐way analysis of variance (ANOVA), followed by Duncan's multiple range test. Differences were considered statistically significant at *p* < 0.05.

## 3 Results

### 3.1 Effect of WFs on the Rheological Properties of Biscuit Dough

The properties of dough, including its texture and handling characteristics—directly influence the taste, appearance, nutritional value, and overall quality of the final biscuit product, serving as key indicators of biscuit quality. To further investigate how dough properties affect biscuit quality, the starch gelatinization behavior in biscuit dough enriched with WFs was evaluated as a function of temperature using dynamic rheological analysis. Key parameters, including the storage modulus (G′), loss modulus (G″), and loss tangent (tan δ = G″/G′), were used to characterize the viscoelastic properties of the dough. A higher G′ relative to G″ indicates a more elastic and structurally robust dough, which is desirable for producing crispy products such as biscuits (Sinthusamran et al. 2019).

**FIGURE 1:**
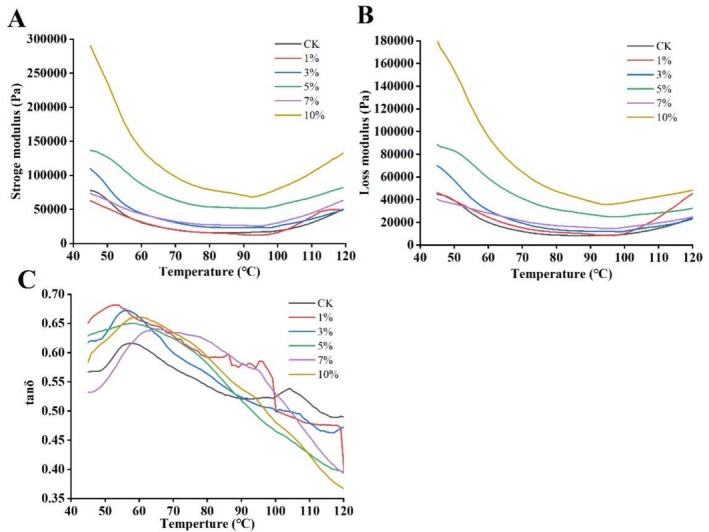
Effect of walnut flowers (WFs) incorporation on the rheological properties of biscuit dough: (A) storage modulus (G′), (B) loss modulus (G″), and (C) loss tangent (tan δ). Control (0% WFs) and samples with WFs added at 1%, 3%, 5%, 7%, and 10% (w/w, flour basis) are shown. G′ reflects elastic behavior, G″ reflects viscous behavior, and tan δ (G″/G′) represents the viscoelastic balance.

The rheological properties of dough with varying WFs content are presented in Figure 1. As the WFs substitution level increased, the storage modulus (G′) of the dough also increased (Figure 1A). For instance, with 5% WFs addition, G′ increased from 500 to 600Pa compared to the control. Similarly, G″ also showed an increasing trend (Figure 1B), suggesting concurrent enhancements in both elastic and viscous components, thereby forming a relatively ideal network structure that meets the textural requirements for crisp biscuits. This behavior may be attributed to interactions between the dietary fiber in WFs and gluten proteins, facilitating the formation of a more compact three‐dimensional network stabilized by hydrogen bonding. Furthermore, the high water‐absorption capacity of the fiber likely contributed to greater internal friction and energy dissipation within the dough matrix.

The temperature dependence of tan δ (Figure 1C) further elucidated the dynamic changes in the viscoelastic balance. An increase in tan δ at 45°C with WFs addition suggested that fiber‐mediated water absorption led to localized network softening, thereby enhancing the viscous contribution. In contrast, tan δ decreased uniformly across all samples at 55°C, likely due to the synergistic effects of starch granule swelling and protein network stabilization, which reinforced elastic‐dominant behavior. All tan δ values remained below 1, confirming that WFs incorporation promoted network elasticity, aligning with the textural requirements for crisp products (Sinthusamran et al. 2019).

### 3.2 Effect of WFs Addition on the Nutritional Properties of Biscuits

The nutritional composition of biscuit samples formulated with varying levels of WFs substitution is summarized in Table 1. WFs contained 23.21 ± 0.06 g/100 g dry weight (DW) crude protein, 46.20 ± 0.15 g/100 g DW total dietary fiber (TDF), and 56.5 ± 1.60 mg/g DW total minerals, thus confirming its potential as a nutrient‐rich ingredient.

**TABLE 1:** Nutritional composition of biscuits containing different percentages of WFs.

Component	WFs (100%)	0% WFs (Control)	1% WFs	3% WFs	5% WFs	7% WFs	10% WFs
Crude Proteins (g/100 g DW)	23.21 ± 0.06^a^	12.00 ± 0.13^b^	11.91 ± 0.09^b^	10.29 ± 0.08^d^	11.21 ± 0.12^c^	10.30 ± 0.12^d^	11.43 ± 0.22^c^
Total sugar (mg/100 g DW)	—	565.89 ± 10.03^b^	581.91 ± 12.91^b^	602.17 ± 19.68^a^	576.99 ± 9.61^b^	545.64 ± 3.65^c^	543.54 ± 7.85^c^
Ash (% DW)	13.10 ± 0.29^a^	0.29 ± 0.03^c^	0.30 ± 0.10^b^	0.31 ± 0.22^c^	0.49 ± 0.26^c^	0.57 ± 0.06^c^	0.76 ± 0.43^c^
Total dietary fiber (g/100 g DW)	46.20 ± 0.15^a^	8.52 ± 1.14^b^	10.40 ± 1.7^b^	8.86 ± 2.41^b^	11.50 ± 3.27^b^	12.55 ± 2.72^b^	12.66 ± 2.99^b^
Soluble carbohydrates (mg/g DW)	56.5 ± 1.60^d^	894.87 ± 47.60^a^	883.41 ± 21.23^a^	844.76 ± 10.14^ab^	818.06 ± 5.38^bc^	805.15 ± 41.41^bc^	778.82 ± 43.76^c^

*Note:* Values are expressed as mean ± standard deviation (*n* = 3). Different superscript letters within a row indicate significant differences (*p* < 0.05). DW: dry weight.

The incorporation of WFs significantly altered the nutritional profile of the biscuits (Table 1). The crude protein content showed a non‐linear response to WFs addition, with significant differences (*p* < 0.05) observed among formulations. For instance, the protein content in the 3% WFs biscuits (10.29 g/100 g DW) was significantly lower than that of the control (12.00 g/100 g DW). This variability may be attributed to the dilution of wheat gluten and the potential for uneven distribution of the WFs during mixing. In contrast, there was a clear increasing trend in the ash content, rising from 0.29% DW in the control to 0.76% DW in the 10% WFs biscuits. Specifically, this increase directly reflects the elevated mineral content contributed by WFs (Contreras‐Jiménez et al. 2019), thereby enhancing the mineral density of the product, which aligns with findings from similar studies using plant by‐products (Han and Lee 2021).

Total dietary fiber (TDF) content also increased significantly from 8.52 to 12.66 g/100 g DW in the control to the 10% WFs formulation (*p* < 0.05), but the TDF levels in the 5%–7% WFs groups were already close to that of the 10% group, indicating that even low doses can effectively improve fiber content. Dietary fiber is recognized for its role in the primary prevention of chronic diseases such as diabetes and obesity. It can modulate starch digestion and absorption by altering its physicochemical environment and encapsulating starch granules (Hua et al. 2023; Wu et al. 2023).

Conversely, both total sugar and soluble carbohydrate contents showed a general decreasing trend with higher WFs substitution levels. The total sugar content decreased from 565.89 to 543.54 mg/100 g DW, while soluble carbohydrates declined from 894.87 mg/g DW to 778.82 mg/g DW in the 10% WFs biscuits. These reductions indicate a lower content of readily available carbohydrates compared to the control. Diets lower in available carbohydrates have been associated with reduced risks of obesity, diabetes, and cardiovascular diseases (Brouns 2018). Therefore, the incorporation of WFs into biscuit formulations provides a nutritionally improved profile with potential metabolic health benefits.

### 3.3 Effect of WFs Addition on Water‐Holding Capacity (WHC) of Biscuits

**FIGURE 2:**
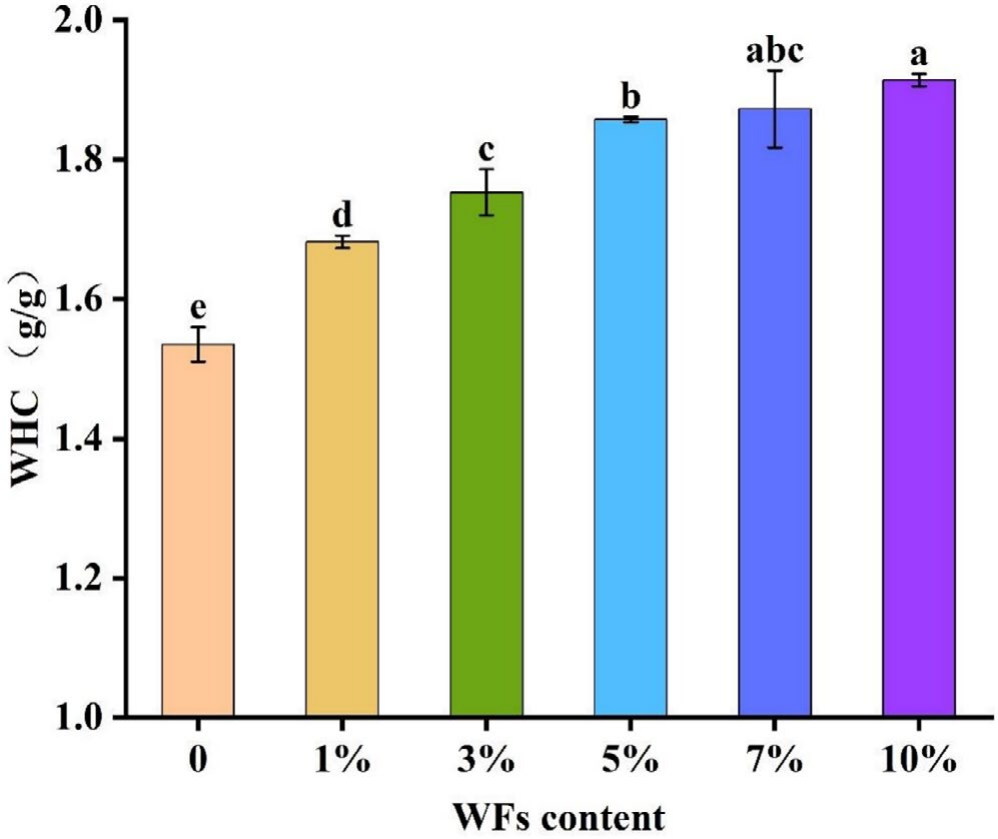
Water‐holding capacity (WHC) of biscuit samples containing different percentages of WFs. Bars with different lowercase letters indicate significant differences between different levels of WFs (*p* < 0.05).

Water‐holding capacity (WHC) is a critical parameter for evaluating the texture, shelf life, and overall quality of biscuits (Watanabe et al. 2018). The influence of WFs incorporation on the WHC of biscuit samples is shown in Figure 2. WFs addition significantly increased the WHC in a concentration‐dependent manner (*p* < 0.05), with the effect plateauing at the 5% substitution level. Higher incorporation rates resulted in excessive water absorption, which compromised the dough's structural integrity. This enhancement can be attributed to the high dietary fiber content and other high‐molecular‐weight components in WFs, which promote moisture absorption and retention (Cao et al. 2024). A similar effect has been reported in bread formulations containing grape seed powder, where increased WHC was linked to the high fiber content and porous microstructure of the powder, facilitating water binding through hydrogen bonding and capillary action (Elkatry et al. 2022). These mechanisms likely contribute to the improved WHC observed in WFs‐fortified biscuits.

### 3.4 Effect of WFs Addition on the Texture of Biscuits

**FIGURE 3:**
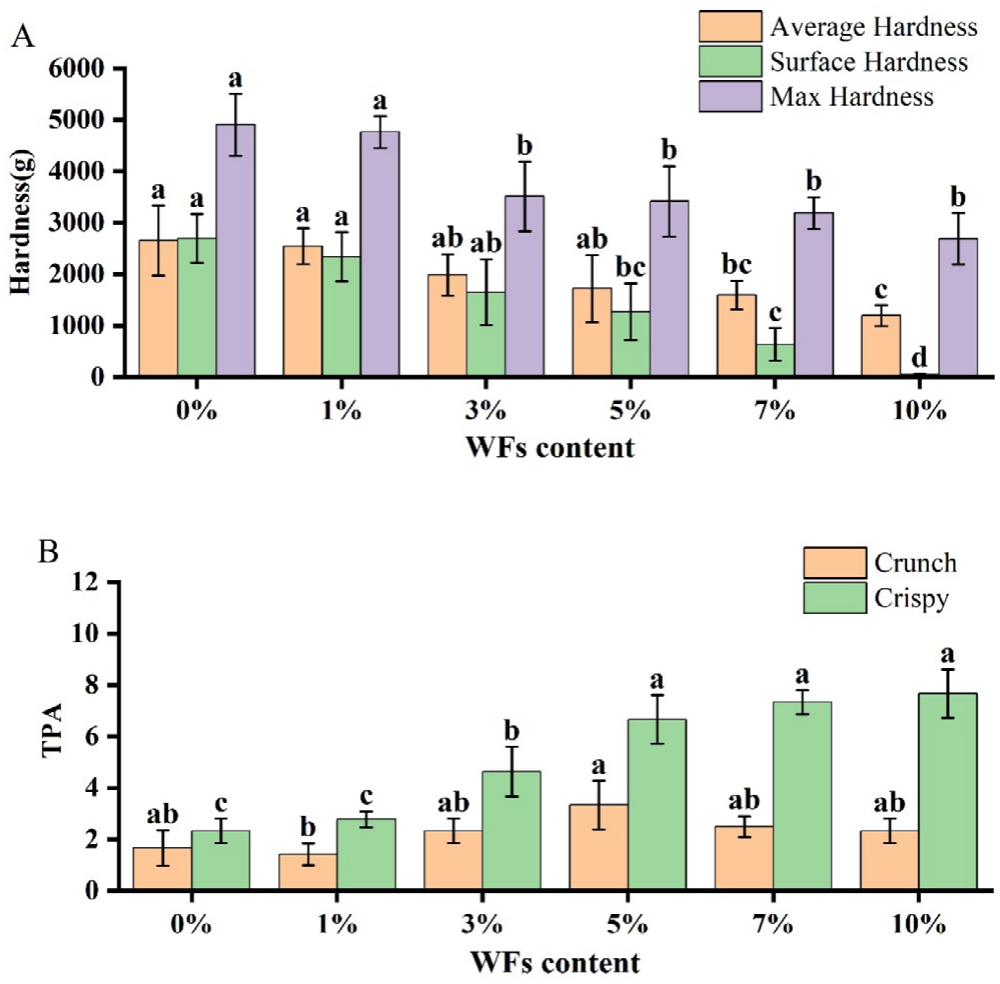
Texture properties of biscuit samples containing different percentages of WFs: (A) hardness and (B) crispness. Bars with different lowercase letters indicate significant differences between different levels of WFs (*p* < 0.05).

For crisp products like biscuits, a reduction in hardness and an enhancement of brittleness are desirable texture attributes (Ma et al. 2023). As shown in Figure 3, increasing the proportion of WFs within the tested range significantly reduced the hardness of the biscuit samples (Figure 3A). For instance, at 10% WFs substitution, the hardness decreased from 50 to 30 N compared to the control. Concurrently, higher WFs levels led to significantly enhanced crispness (Figure 3B).

The observed texture modifications can be attributed to the high dietary fiber content in WFs, which interferes with gluten network formation and promotes a more porous and fragile structure (Wang et al. 2014). Furthermore, phenolic compounds present in WFs have been shown to inhibit gluten cross‐linking, resulting in a less rigid matrix and reduced hardness (Al‐Marazeeq et al. 2024). These findings align with previous reports on the role of dietary fibers in altering the texture of baked goods by disrupting gluten continuity and increasing porosity (Zhang et al. 2017).

### 3.5 Effect of WFs Addition on the Microstructure of Biscuits

**FIGURE 4:**
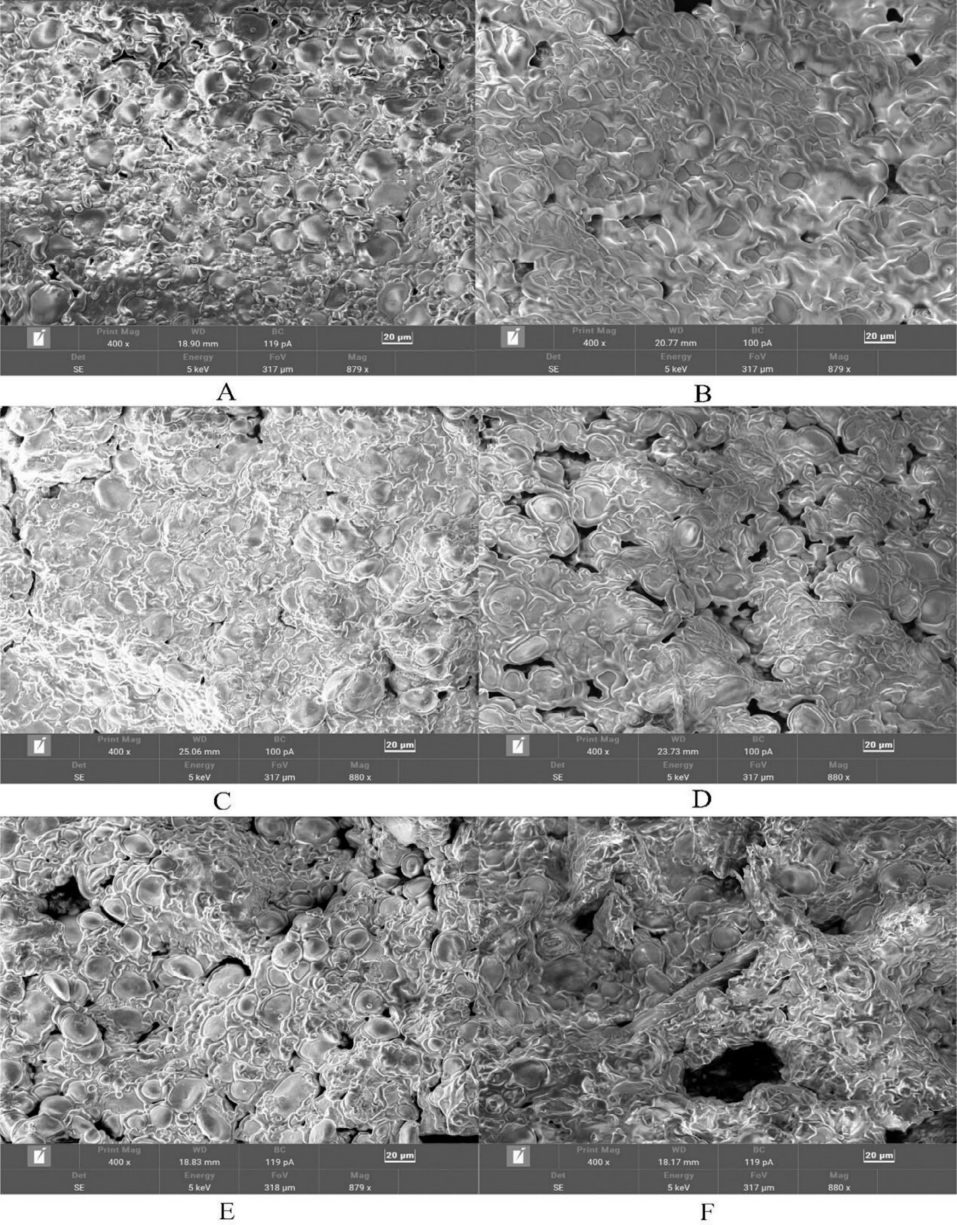
Scanning electron microscopy (SEM) micrographs (400× magnification) of biscuit samples containing different percentages of WFs: (A) 0% (control), (B) 1%, (C) 3%, (D) 5%, (E) 7%, and (F) 10%.

Scanning electron microscopy (SEM) is a well‐established technique for examining the microstructure of baked products such as biscuits (Alongi et al. 2019). SEM micrographs revealed distinct structural differences between formulations. The control biscuit (0% WFs) exhibited a uniform and dense gluten network with a compact matrix (Figure 4A). As the level of WFs incorporation increased from 3% to 10%, the biscuit samples displayed progressively larger and more numerous voids, indicating a more open and discontinuous structure (Figure 4B–F).

Dietary fibers have been shown to modify the microstructure of baked goods by physically occupying space within the matrix, competing with gluten proteins for water, and encapsulating starch granules (Jia et al. 2020). In the present study, the increasing WFs content likely disrupted the interaction between gliadin and glutenin, leading to a discontinuous gluten network and the formation of larger pores. Overall, combined analysis of the texture and specific mechanical energy (SME) of the biscuits indicated that at a WF substitution level of 5%–7%, the textural properties and gluten network could be maintained at a relatively optimal level. However, at a 10% substitution level, excessive weakening of the gluten structure may occur, resulting in textural deterioration.

### 3.6 Effect of WFs Addition on the Glycemic Response During in Vitro Starch Digestion

**FIGURE 5:**
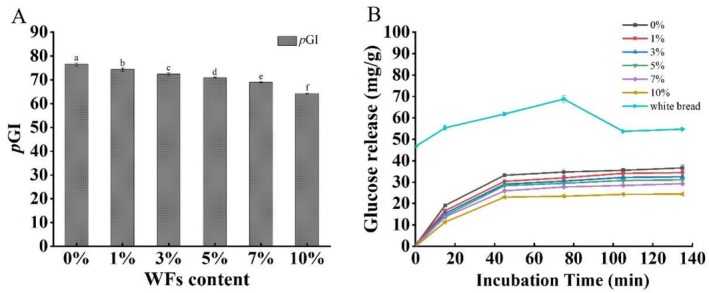
Effect of WFs addition on (A) predicted glycemic index (*p*GI) values and (B) in vitro glucose release profiles during starch digestion. Bars with different lowercase letters indicate significant differences among samples (*p* < 0.05).

The release of carbohydrates during the simulated digestion of walnut male flowers (WFs)‐enriched biscuits reflects their potential postprandial blood glucose response, commonly assessed via the glycemic index (GI). As shown in Figure 5A, the predicted glycemic index (*p*GI) of biscuits significantly decreased with increasing WFs substitution (*p* < 0.05). Specifically, the *p*GI of biscuits with 10% WFs decreased to 64.16, representing a 16% reduction compared to the control (*p*GI = 76.42). This result confirms that WFs incorporation effectively lowers the GI of biscuits.

Additionally, the glucose release kinetics during digestion are presented in Figure 5B. Glucose release from all biscuit samples increased over time. However, at each time point, the amount of released glucose was inversely related to the level of WFs incorporation. In the control sample (0% WFs), the final glucose concentration reached 47.59 mg/g. With 10% WFs addition, this value decreased significantly to 24.37 mg/g, indicating a strong inhibitory effect of WFs on carbohydrate digestion.

The reduction in *p*GI and delayed glucose release can be attributed to bioactive components present in WFs. The high dietary fiber content can encapsulate starch granules and limit their accessibility to digestive enzymes, thereby delaying hydrolysis (Jia et al. 2020). Furthermore, phenolic compounds in WFs have been shown to inhibit α‐glucosidase activity, slowing the enzymatic conversion of carbohydrates into glucose (Ahmad et al. 2020). These mechanisms collectively contribute to the slower starch hydrolysis and reduced glycemic response, supporting the potential of WFs in developing low‐GI functional foods.

### 3.7 Effect of WFs Addition on the Color and Sensory Properties of Biscuits

The influence of WFs incorporation on the surface color of biscuit samples is summarized in Table 2. The control biscuit (0% WFs) exhibited the highest values for lightness (L*), yellowness (b*), chroma (C*), and hue angle (h°), while the redness value (a*) was the lowest. As the WFs substitution level increased from 0% to 10%, the L* value decreased significantly from 75.37% to 44.00%. This darkening effect was likely due to polyphenol oxidase (PPO) activity in WFs and the promotion of melanoidin formation during the Maillard reaction (Pycia et al. 2022). Concurrently, a* values increased significantly, indicating a shift toward redder hues. The gradual decreases in b* and C* values suggested a less intense yellow color, while the decline in h° from 84.65 to 61.86 reflected an overall color shift from yellow toward reddish‐brown tones. Based on the color parameters, visual acceptability was optimal at the 5% WFs substitution level.

**TABLE 2:** Color parameters of biscuits containing different percentages of WFs.

Parameter	0% WFs	1% WFs	3% WFs	5% WFs	7% WFs	10% WFs
L*	75.37 ± 0.59^a^	66.91 ± 1.27^b^	54.58 ± 0.6^c^	52.80 ± 2.12^d^	47.70 ± 0.46^e^	44.00 ± 0.28^f^
a*	2.46 ± 0.07^a^	4.07 ± 0.20^b^	6.32 ± 0.004^c^	6.39 ± 0.57^d^	5.95 ± 0.13^e^	6.58 ± 0.09^f^
b*	26.3 ± 0.14^a^	20.20 ± 0.27^b^	16.55 ± 0.02^c^	15.63 ± 0.87^d^	13.67 ± 0.37^e^	12.35 ± 0.66^f^
c*	26.41 ± 0.13^a^	20.60 ± 0.30^b^	17.71 ± 0.04^c^	16.91 ± 0.96^d^	14.91 ± 0.40^e^	14.01 ± 0.62^f^
h*	84.65 ± 0.18^a^	78.62 ± 0.41^b^	69.09 ± 0.11^c^	67.69 ± 1.44^d^	66.46 ± 0.16^e^	61.86 ± 0.97^f^

*Note:* Values are mean ± standard deviation (*n* = 3). Different lowercase superscript letters within a row indicate significant differences (*p* < 0.05).

**FIGURE 6:**
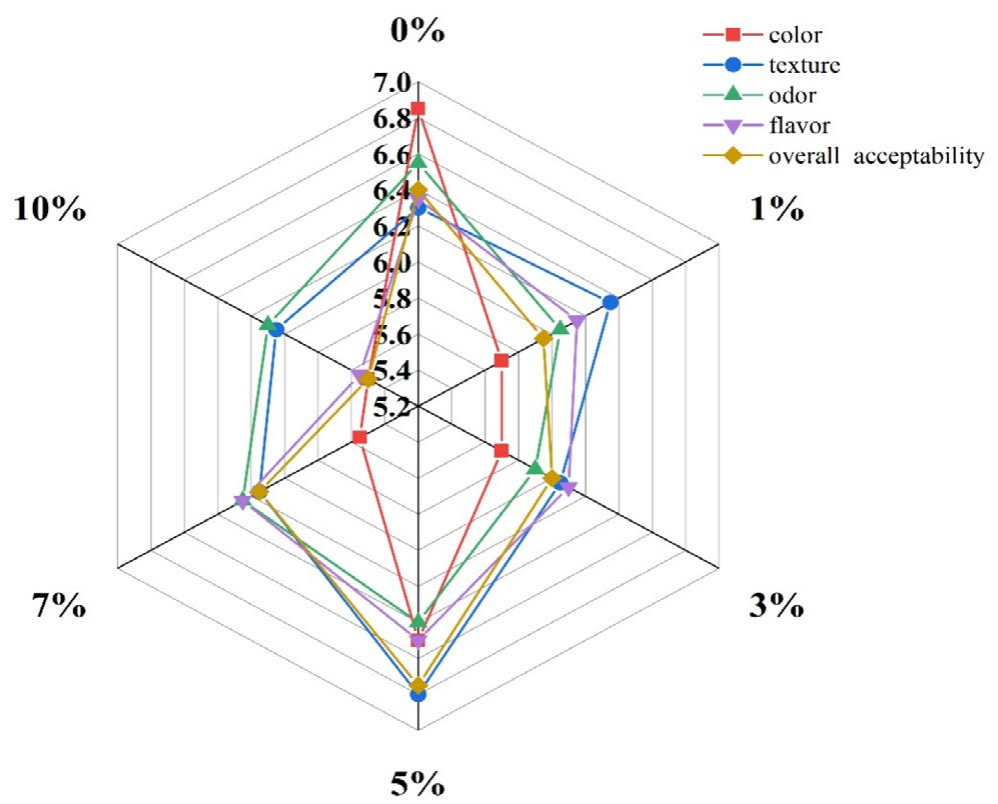
Sensory evaluation scores of biscuits formulated with different percentages of WFs.

Sensory evaluation results are presented in Figure 6. Biscuits containing 5% WFs received the highest overall acceptability score (6.75 on the 9‐point hedonic scale), compared to 5.5 for the control. For texture, samples with 5% WFs scored higher than those with lower substitution levels. However, WFs levels above 5% adversely affected perceived moisture, hardness, and mouthfeel, increasing grittiness and astringency (Miller and Chambers 2013), which led to reduced texture scores. A similar trend was observed for flavor and aroma attributes, where scores increased up to 5% WFs and then declined at higher levels. Color scores were highest for the 5% WFs formulation, which aligns with the instrumental color measurements (Table 2).

In summary, biscuits formulated with 5% WFs received the highest scores for key sensory attributes, including texture (6.8), aroma (6.4), mouthfeel (6.5), and overall acceptability (6.75). These samples were characterized by a reddish‐brown color, a crisp yet tender texture, and a pleasant, distinctive flavor and aroma attributed to the WFs' components.

## 4 Discussion

This study demonstrates that WFs serve as a functional ingredient capable of synergistically improving the processing properties, nutritional quality, and sensory acceptability of biscuits, thereby addressing the growing market demand for healthier baked goods.

Rheological analysis revealed that WFs incorporation significantly increased the storage modulus (*G*′) and loss modulus (*G*″) of the dough while reducing the loss tangent (tan *δ*) at elevated temperatures. This indicates enhanced viscoelasticity and a stabilized network structure, which facilitates the formation of a crisp texture. These findings align with the mechanism by which fiber‐rich by‐products (e.g., apple pomace) reinforce dough stability (Alongi et al. 2019), primarily attributed to dietary fiber‐gluten interactions forming a denser network. Variations in efficacy among fiber sources warrant further investigation to refine strategies for modulating dough properties.

Nutritionally, WFs significantly elevated the protein, ash, and total dietary fiber (TDF) content while reducing total sugar and soluble carbohydrate. A 10% substitution achieved a maximum 16% reduction in predicted glycemic index (*p*GI), consistent with reports on dietary fiber in regulating starch digestibility (Brouns 2018) and functional ingredients mitigating postprandial glucose response (Elkatry et al. 2022). The underlying mechanisms likely involve physical encapsulation of starch granules by dietary fibers and inhibition of α‐glucosidase activity by phenolic compounds. Elucidating WFs‐specific nutrient interactions and component synergies remains crucial for formula optimization.

Texture properties exhibited dose dependency: Increasing WFs levels reduced hardness and enhanced crispiness, with microstructural analysis confirming larger voids and more open networks—consistent with fiber‐induced gluten network disruption for optimal texture (Jia et al. 2020). However, exceeding 5% substitution impaired texture due to increased roughness and astringency. This threshold is modulated by particle size and fiber characteristics, necessitating further study to expand applicable dosage ranges.

Sensory evaluation identified 5% as the optimal level, balancing traditional biscuit attributes with WFs' distinctive nutty aroma, Maillard reaction‐derived reddish‐brown hue, and enzymatic browning. This validates the principle that moderate incorporation of fruit/vegetable by‐products enhances sensory profiles without compromising acceptability (Bora et al. 2019), though consumer preference mapping for distinct flavor notes requires deeper exploration.

In summary, WFs improve biscuit quality through mechanisms shared with analogous fiber sources while highlighting unique value as a walnut‐derived by‐product. Our multidimensional analysis establishes a foundation for developing nutritionally enhanced, sensorially appealing products. Future research should prioritize comparative mechanistic evaluation of diverse fiber sources, synergistic interactions among key constituents in WFs, elucidation of textural determinants, and evidence‐based guidance for consumer sensory preferences.

## 5 Conclusion

The incorporation of WFs into biscuit formulations synergistically enhanced the functional properties of the product, primarily through the combined contributions of its dietary fiber and phenolic compounds. These components modified the dough's rheological behavior, promoting a more elastic network that translated into a crispier texture in the baked biscuits. Nutritionally, WFs enrichment significantly improved the product profile by increasing the ash and total dietary fiber content while reducing available carbohydrates and sugar levels. These changes contributed to a notably lower predicted glycemic index (*p*GI), highlighting the potential of WFs‐fortified biscuits for moderated postprandial glucose responses. Sensory evaluation identified 5% (w/w, flour basis) as the optimal substitution level, with biscuits at this concentration receiving the highest overall acceptability. These samples successfully integrated the distinctive, pleasant aroma and flavor notes of WFs with the familiar qualities of conventional biscuits. Based on a comprehensive evaluation of the biscuit texture, nutritional properties, processing performance, and sensory assessment, it was determined that the addition of 5% WFs resulted in optimal nutritional value, glycemic index (GI), sensory scores, and processing characteristics.

In summary, this study provides a scientific basis for developing nutritious, low‐glycemic‐index baked goods and supports the valorization of WFs as a sustainable, functional food ingredient. However, this study has certain limitations, particularly the insufficient investigation of flavor and metabolite changes in biscuits following the addition of WFs. Future research should focus on analyzing variations in the volatile and non‐volatile components of WFs‐enriched biscuits to fully elucidate the impact of WFs on their flavor profile and functional properties. The findings encourage the utilization of similar agricultural by‐products, promoting resource efficiency and adding value to food production systems.

## Author Contributions

**Guoshuang Qin:** methodology, formal analysis, investigation, writing – original draft. **Yajing Li:** methodology, investigation, formal analysis, data curation. **Jian Sun:** investigation, validation, writing – review and editing. **Zhenxing Wang:** software, resources, validation, writing – review and editing. **Jinggui Nie:** investigation, validation, writing – review and editing. **Kin Weng Kong:** validation, writing – review and editing. **Amin Ismail:** validation, writing – review and editing. **Yongyu Si:** validation, supervision, funding acquisition, writing – review and editing. **Xuechun Zhang:** conceptualization, funding acquisition, project administration, resources, validation, writing – review and editing.

## Ethics Statement

The sensory evaluation involving human participants was reviewed and approved by the Ethics Committee of Southwest Forestry University (Approval No. SWFU‐2023026). Informed consent was obtained from all participants prior to the study.

## Conflicts of Interest

The authors declare no conflicts of interest.

## Data Availability

The data that support the findings of this study are available from the corresponding author upon reasonable request.
